# Hospital factor and prognosis of COVID-19 in New York City, the United States of America: insights from a retrospective cohort study

**DOI:** 10.1186/s12913-022-07570-w

**Published:** 2022-02-08

**Authors:** Mai Takahashi, Natalia N. Egorova, Masao Iwagami, Toshiki Kuno

**Affiliations:** 1 Department of Medicine, Icahn School of Medicine at Mount Sinai, Mount Sinai Beth Israel, New York, NY USA; 2 Department of Population Health Science and Policy, Icahn School of Medicine at Mount Sinai, New York, USA; 3 Department of Health Services Research, University of Tsukuba, Tsukuba, Japan; 4 Division of Cardiology, Montefiore Medical Center, Albert Einstein College of Medicine, 111 East 210th St, New York, NY 10467–2401 USA

**Keywords:** COVID-19, Hospital factor, Teaching hospital, Community hospital

## Abstract

**Background:**

April 22nd, 2020, New York City (NYC) was the epicenter of the pandemic of Coronavirus disease 2019 (COVID-19) in the US with differences of death rates among its 5 boroughs. We aimed to investigate the difference in mortality associated with hospital factors (teaching versus community hospital) in NYC.

**Design:**

Retrospective cohort study.

**Methods:**

We obtained medical records of 6509 hospitalized patients with laboratory confirmed COVID-19 from the Mount Sinai Health System including 4 teaching hospitals in Manhattan and 2 community hospitals located outside of Manhattan (Queens and Brooklyn) retrospectively. Propensity score analysis using inverse probability of treatment weighting (IPTW) with stabilized weights was performed to adjust for differences in the baseline characteristics of patients initially presenting to teaching or community hospitals, and those who were transferred from community hospitals to teaching hospitals.

**Results:**

Among 6509 patients, 4653 (72.6%) were admitted in teaching hospitals, 1462 (22.8%) were admitted in community hospitals, and 293 (4.6%) were originally admitted in community and then transferred into teaching hospitals. Patients in community hospitals had higher mortality (42.5%) than those in teaching hospitals (17.6%) or those transferred from community to teaching hospitals (23.5%, *P* < 0.001). After IPTW-adjustment, when compared to patients cared for at teaching hospitals, the hazard ratio (HR) and 95% confidence interval (CI) of mortality were as follows: community hospitals 2.47 (2.03-2.99); transfers 0.80 (0.58-1.09)).

**Conclusions:**

Patients admitted to community hospitals had higher mortality than those admitted to teaching hospitals.

**Supplementary Information:**

The online version contains supplementary material available at 10.1186/s12913-022-07570-w.

## Introduction

Coronavirus disease 2019 (COVID-19) caused by a novel coronavirus, severe acute respiratory syndrome coronavirus 2 (SARS-CoV2), has spread globally since the first cases were reported in December 2019 [1]. The World Health Organization declared COVID-19 to be a pandemic on March 11, 2020, and as of April 22nd, New York City had become the epicenter of the pandemic in the US [2]. On Apr 26th, 2021, the number of deaths due to the COVID-19 pandemic exceeded 3 million globally and the total number of cases exceeded 140 million [2], with more than 30 million in the US alone. Notably, on May 2nd, 2020, New York State had 310,000 confirmed cases surpassing any other country including Spain, Italy, and China [2]. New York City saw the number of confirmed COVID-19 cases rise at an astounding rate, with its first known case on February 29th, 2020, and the first confirmed death on March 14th, 2020. Since then, the total number of deaths has risen exponentially to 12,895, with 172,354 patients suffering from COVID-19 as of May 2nd, with the higher rates of COVID-19 in two boroughs: Queens and Brooklyn [2]. Hospitals and healthcare systems tried to adapt to the rapid pace at which this unprecedented viral illness was spreading. However, hospital care and supply, including human resources, have fallen short - especially during the initial phase of the pandemic in suburban areas, resulting in substantially different mortality rates in the 5 New York City boroughs [3]. Our hypothesis was that these differences might be partially explained by hospital factors such as teaching versus community status.

The aim of this study was to investigate the difference in mortality associated with hospital factors (teaching status) in New York City in the first wave of the COVID-19 pandemic.

## Methods

This retrospective study was conducted using medical records of patients hospitalized with laboratory confirmed COVID-19 in the Mount Sinai Health system between March 1st, 2020, and May 7th, 2020. Hospitals were categorized as teaching hospitals, all of which are located in Manhattan (Mount Sinai Hospital (1134 beds), Mount Sinai West (514 beds), Mount Sinai Morningside (495 beds), and Mount Sinai Beth Israel (701 beds), and community hospitals which are located outside of Manhattan (Mount Sinai Queens hospital (235 beds), and Mount Sinai Brooklyn (212 beds)). As of May 1st, all hospitals have exceeded the pre-COVID-19 maximum capacity of intensive care unit (ICU) beds (May 1st ICU bed numbers (pre-COVID-19 ICU bed max)); Mount Sinai Hospital: 252 (174) beds, Mount Sinai West: 96 (90) Mount Sinai Morningside: 88 (50) beds, Mount Sinai Beth Israel: 77 (58) beds, Mount Sinai Queens: 52 (25) beds, Mount Sinai Brooklyn: 27 (12) beds. Identification of COVID-19 required a polymerase chain reaction test on a sample obtained with a nasopharyngeal swab. The decision to admit the patient was largely provider dependent, and not based on any specific predetermined criteria since very little was known about the disease. Agency for Healthcare Research and Quality (AHRQ)‘s database on Social Determinants on Health (SDOH) was used to estimate the social determinants of patients in New York City by patient residency zip code.

### Cohort description

Patients’ electronic medical records were used to obtain information on demographics, clinical course, comorbidities, and clinical outcomes. Patients were included into 3 mutually exclusive groups: 1) those admitted to teaching hospitals, 2) those admitted to community hospitals, and 3) those who were transferred from community to teaching hospitals. A hospital is considered a teaching hospital if it satisfies one or more of the following criteria: 1) is an Accreditation Council for Graduate Medical Education approved residency program, 2) is a member of the Council of Teaching Hospitals or 3) has a ratio of full-time equivalent interns and residents to beds of .25 or higher. All patients included in this study had a known clinical outcome as of May 7th, 2020.

Differences in baseline characteristics between groups were evaluated using analysis of variance for continuous variables and the χ^2^ test for categorical variables. The Bonferroni correction was used to adjust for multiple comparisons. Continuous variables were presented as mean ± standard deviation or median (interquartile range), and categorical variables were expressed as percentages. All vital signs were recorded at the time of admission. Comorbidities were characterized using the Elixhauser comorbidity method based on the International Classification of Disease (ICD) 10 codes. The primary outcome of interest was in-hospital mortality. Survival curves were constructed using the Kaplan-Meier method and the pairwise comparison was done with Sidak adjustment for multiple comparisons. To adjust for the differences between patients’ groups (teaching, community, and transfer) in the time-to-event analysis, three Cox models were fitted [1]. The transfer was modeled as a time-varying covariate. We fitted the Cox proportional hazard model where the outcome was in-hospital mortality with covariates including the location of care (teaching hospitals versus community hospitals or transfer), patient age, sex, race, body mass index (BMI) (normal [BMI 18.5-25 kg/m^2^] versus overweight [BMI 25-30 kg/m^2^] or obese [BMI > 30 kg/m^2^]) [4], mean values of largest recorded vitals during admission (temperature, heart rate, respiratory rate, systolic blood pressure, diastolic blood pressure, and oxygen saturation level), asthma, chronic obstructive pulmonary disease (COPD), hypertension, obesity, diabetes mellitus, chronic kidney disease, human immunodeficiency virus (HIV), cancer, atrial fibrillation, heart failure, alcoholic/non-alcoholic liver disease, median household income, median number of people in a household, and the median percentage of people with less than high school education (ages 25 and over) by zip code [2]. A propensity score was calculated using a multinomial regression model. The independent variables are shown in Supplemental List 1. Cox proportional hazard models that included inverse propensity treatment weight (IPTW) adjustments were used to analyze the effect of a hospital factor on in-hospital mortality [3]. As a sensitivity analysis, we added endotracheal intubation into the Cox proportional hazard models. We also compared in-hospital mortality between teaching hospitals and combined community hospital/transfers status using the Cox proportional hazard models that included IPTW adjustments [4]. Variables used to estimate the propensity score can be found in Supplemental List 2. All statistical calculations and analyses were performed using R software (version 3.6.2, R Foundation for Statistical Computing, Vienna, Austria), with *p*-values < 0.001 considered statistically significant.

This study was approved by the Institutional Review Board (#2000495) at the Icahn School of Medicine at Mount Sinai and conducted under with the principles of the Declaration of Helsinki. The waiver of patients’ informed consent was also approved by the institutional review boards.

### Patient and public involvement

There was no patient or public involvement in the study design, data analysis, or interpretation of the study results.

## Results

Of the 6509 patients admitted due to COVID-19, 6408 (98.4%) were from New York City. Among them, 4653 (72.6%) were cared for at teaching hospitals, 1462 (22.8%) at community hospitals, and 293 (4.6%) patients were transferred from community to teaching hospitals. Baseline characteristics and vital signs across study groups are reported in Table 1. Patients admitted to community hospitals were generally older, more likely to be black, and had a higher prevalence of COPD, hypertension, diabetes mellitus, chronic kidney disease, atrial fibrillation, and heart failure. Patients admitted to teaching hospitals were more likely to have asthma, obstructive sleep apnea, HIV, cancer, alcoholic/non-alcoholic liver disease. Patients transferred from community to teaching hospitals were younger than those treated in community or teaching hospitals and more likely to be male (Table 1). Notably, respiratory rates were significantly higher among patients in teaching hospitals and those with the transfer. Oxygen saturation levels were significantly lower in patients with transfer than among patients in two other groups.

**Table 1:** Baseline characteristics of patients admitted to teaching, community hospitals and transferred from community to teaching hospitals

	Teaching hospitals (*n* = 4653)	Community hospitals (*n* = 1462)	Transfer (*n* = 293)	*P*-Value
Age, (mean, SD), year	61.4 (18.0)	69.7 (14.8)	59.8 (15.4)	< 0.001
18-44, n (%)	945 (19.9)	83 (5.6)	54 (18.2)	
45-64	1586 (33.4)	431 (29.3)	124 (41.9)	
65 and older	2211 (46.6)	957 (65.1)	118 (39.9)	
Female, n (%)	2142 (46.0)	614 (42.0)	99 (33.8)	< 0.001
Race, n (%)				< 0.001
White	999 (21.5)	407 (27.8)	55 (18.8)	
Black	1073 (23.1)	437 (29.9)	77 (26.3)	
Hispanic	1366 (29.4)	303 (20.7)	93 (31.7)	
Asian	204 (4.4)	81 (5.5)	17 (5.8)	
Other	1011 (21.7)	234 (16.0)	51 (17.4)	
Smoking History, n (%)				< 0.001
Never	2196 (47.2)	856 (58.5)	178 (60.8)	
Not Asked	123 (2.6)	127 (8.7)	11 (3.8)	
Passive	4 (0.1)	0 (0)	0 (0)	
Quit	980 (21.1)	274 (18.7)	44 (15.0)	
Yes	218 (4.7)	45 (3.1)	15 (5.1)	
Asthma, n (%)	272 (5.9)	46 (3.2)	11 (3.8)	< 0.001
COPD, n (%)	167 (3.6)	69 (4.7)	6 (2.0)	0.042
Hypertension, n (%)	1441 (31.2)	535 (36.7)	90 (30.7)	< 0.001
Obstructive Sleep Apnea, n (%)	121 (2.6)	11 (0.8)	1 (0.3)	< 0.001
Diabetes mellitus, n (%)	933 (20.2)	368 (25.2)	60 (20.5)	< 0.001
Chronic Kidney Disease, n (%)	481 (10.4)	190 (13.0)	22 (7.5)	0.003
HIV, n (%)	94 (2.0)	11 (0.8)	3 (1.0)	0.003
Cancer, n (%)	417 (9.0)	58 (4.0)	11 (3.8)	< 0.001
Atrial fibrillation, n (%)	248 (5.4)	123 (8.4)	22 (7.5)	< 0.001
Heart Failure, n (%)	296 (6.4)	117 (8.0)	14 (4.8)	0.043
Alcoholic/Non-alcoholic liver disease, n (%)	111 (2.4)	22 (1.5)	3 (1.0)	0.046
BMI, n (%)				0.071
Underweight or Normal weight	1826 (39.2)	585 (40.0)	92 (31.4)	
Overweight	1304 (28.0)	408 (27.9)	87 (29.7)	
Obese	1523 (32.7)	469 (32.1)	114 (38.9)	
Temperature, (median, IQR)	99.1 (98.5-99.9)	98.8 (98.2-99.5)	99.1 (98.6-100.1)	< 0.001
Heart Rate, (median, IQR)	95.0 (86.4-105.0)	96.5 (87.4-107.3)	99.3 (89.6-110.4)	< 0.001
Respiratory Rate, (median, IQR)	20.0 (19.1-23.5)	19.8 (18.9-24.1)	23.0 (19.7-29.8)	< 0.001
Systolic Blood Pressure, (median, IQR)	137 (126-149)	138 (126-149)	137 (128-147)	0.910
Diastolic Blood Pressure, (median, IQR)	79.2 (74.0-85.0)	77.9 (72.5-83.3)	78.2 (74.0-83.6)	< 0.001
O_2_ Saturation, (median, IQR)	93.3 (91.1-95.7)	93.0 (90.0-95.3)	91.8 (90.0-93.6)	< 0.001
Median Household Income, $ (median, IQR)	54,121 (35859-85,930)	64,067 (56383-71,437)	65,098 (57010-71,437)	0.101
Median Household size, (N of people) (median, IQR)	2.4 (2.1-2.8)	2.7 (2.3-3.0)	2.6 (2.3-3.0)	0.315
Median Percentage of Population with less than High School Education, (median, IQR)	17.6 (10.0-25.7)	14.0 (11.1-19.1)	14.0 (11.8-19.1)	0.234

*BMI* body mass index, *COPD* chronic obstructive pulmonary disease, *HIV* human immunodeficiency virus, *SD* standard deviation

We observed significantly higher mortality rates for patients admitted to community hospitals (45.6%) when compared to those admitted to teaching hospitals (20.2%) or those transferred from community hospitals to teaching hospitals (28.4%) (*P* < 0.001) (Supplemental Table 1). ICU admissions and intubation rates were significantly higher among patients who were transferred than among those cared for at either teaching or community hospitals (Supplemental Table 1).

The difference in death rates by place of care stratified by race is presented in Supplemental Table 2. Notably, half of the white patients in community hospitals died, but they were significantly older than those admitted to teaching hospitals or those who were transferred (Supplemental Tables 2, 3).

**Fig. 1:**
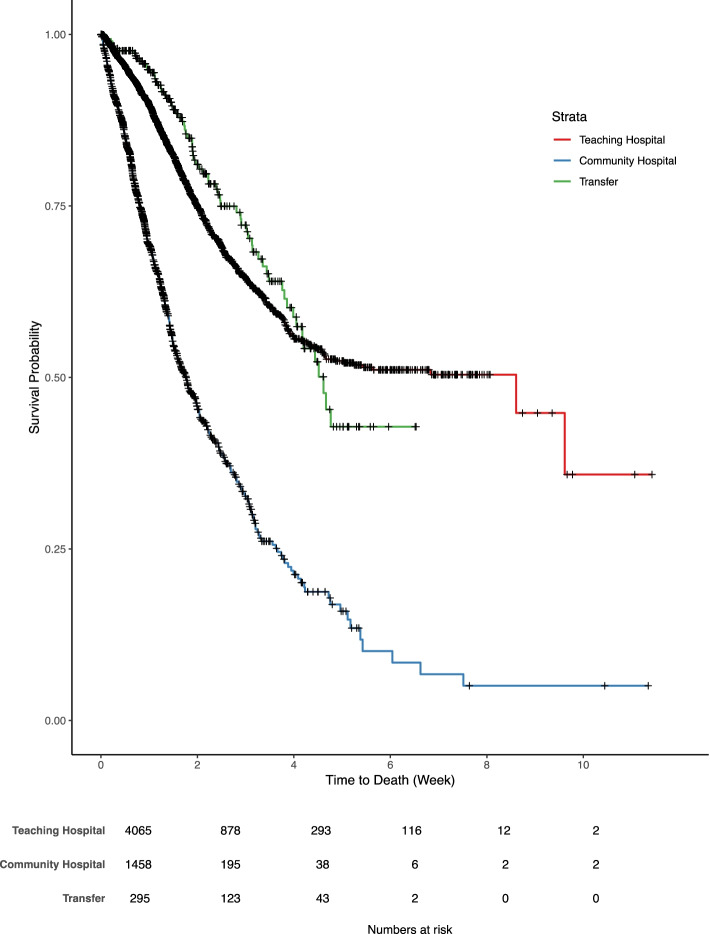
Kaplan-Meier analysis of in-hospital mortality for patients treated in teaching, community hospitals and patients transferred from teaching to community hospitals

The Kaplan Meier analysis showed different survival among patients in the community and teaching hospitals (*P* < 0.001) (Fig. 1). However, there was no difference in survival between patients admitted to teaching hospitals and those who were transferred (*P* = 0.15).

Unadjusted in-hospital mortality was significantly higher in patients admitted to community hospitals (hazard ratio [HR] (95% confidential interval [CI]): 2.97 (2.66–3.31)) (Table 2). After multivariable-adjustment with age, sex, race, body mass index, comorbidities (asthma, COPD, hypertension, obstructive sleep apnea, diabetes, chronic kidney disease, cancer, atrial fibrillation, heart failure,) and vital signs at admission (temperature, heart rate, respiratory rate, systolic blood pressure, diastolic blood pressure, O2 saturation), patients admitted to community hospitals had a significantly higher risk of death than those admitted to teaching hospitals (hazard ratio [HR] (95% confidential interval [CI]) for Model 2: 2.50 (2.22–2.81)) (Table 2). A similar result was obtained when the model was adjusted for variables included in Model 2 and neighborhood characteristics (median house income, median number of people by household, median percentage of population with less than high school education) (teaching hospital versus community hospital, hazard ratio [HR] (95% confidential interval [CI]): 2.56 (2.25–2.92) (Table 2). In-hospital mortality among transferred patients was not significantly different when compared to those admitted to teaching hospitals in either Model 2 or Model 3.

**Table 2:** Unadjusted and Adjusted in-hospital death for patients admitted with COVID-19 based on the results of Cox proportional hazard models

	Model 1: Unadjusted	Model 2: Adjusted for patient characteristics	Model 3: Adjusted for patient and neighborhood characteristics
	HR (95% CI)	HR (95% CI)	HR (95% CI)
Teaching Hospital	Reference	Reference	Reference
Community Hospital	2.97 (2.66 – 3.31)	2.50 (2.22 – 2.81)	2.56 (2.25 – 2.92)
Transferred	0.85 (0.67 – 1.09)	0.84 (0.65 – 1.08)	0.83 (0.64 – 1.08)

*HR* hazard ratio, *CI* confidential interval

Model 2: Adjusted for patient age, sex, race, body mass index, comorbidities (Asthma, COPD, Hypertension, Obstructive Sleep Apnea, Diabetes, Chronic Kidney Disease, Cancer, Atrial Fibrillation, Heart Failure,) and vital signs at admission (Temperature, Heart Rate, Respiratory Rate, Systolic Blood Pressure, Diastolic Blood Pressure, O2 Saturation)

Model 3: Adjusted for variables included in Model 2 and neighborhood characteristics (Median House Income, Median Number of People by Household, Median Percentage of Population with less than High School Education)

After IPTW-adjustments, baseline characteristics of patients were well-balanced (Supplemental Fig. 1, SMDs> 0.20 are considered as potentially important imbalances). Analysis of outcomes after adjustment by IPTW showed that patients admitted to community hospitals had significantly higher mortality than those admitted to teaching hospitals (HR (95% CI): 2.23 (1.85–2.64), *P* < 0.001) (Table 3). Patients transferred from community to teaching hospitals had a lower risk of death when compared to patients who were admitted to community hospitals despite higher rates of ICU admission and endotracheal intubation (HR (95% CI): 0.35 (0.24-0.45), *P* < 0.001). Similar results were seen after adding intubation into the Cox model (Table 3). In-hospital mortality was significantly different between teaching hospitals and combined community hospital/transfers with the Cox proportional hazard models that included IPTW adjustments (HR (95%CI: 1.74 (1.45–2.08, *P* < 0.001) (Table 4).

**Table 3:** Site of care as a risk factor associated with in-hospital mortality. The results based on propensity analysis using Cox proportional hazard models adjusted for inverse probability treatment weights

	HR (95% CI)	*P*-Value
Cox model without Intubation		
Location of Care		
Teaching hospitals	Reference	Reference
Community hospitals	2.47 (2.03 – 2.99)	< 0.001
Transfer	0.80 (0.58 – 1.09)	0.15
Cox model with Intubation		
Location of Care		
Teaching hospitals	Reference	Reference
Community hospitals	2.46 (2.03 – 2.99)	< 0.001
Transfer	0.78 (0.57 – 1.07)	0.12
Intubation	1.31 (1.06 – 1.61)	0.013

*CI* confidential interval, *HR* hazard ratio

**Table 4:** In-hospital mortality among patients receiving care in teaching hospitals versus patients transferred or receiving care in community hospital. The results based on propensity analysis using Cox proportional hazard models adjusted for inverse probability treatment weights

	HR (95% CI)	*P*-Value
Location of Care		
Teaching hospitals	Reference	Reference
Community hospitals/ Transfer	1.74 (1.45 – 2.08)	< 0.001

*CI* confidential interval, *HR* hazard ratio

## Discussion

In a large retrospective cohort study of over 6000 patients with laboratory confirmed COVID-19 during the initial surge in NYC, USA, we found that patients admitted to community hospitals had higher rates of in-hospital mortality than those admitted to teaching hospitals and this difference persisted after multivariable and propensity score adjustments. In addition, patients who were transferred from a community hospital to a teaching hospital had a lower risk of death compared to patients who were cared for exclusively at community hospitals.

New York City is composed of five boroughs (the Bronx, Brooklyn, Manhattan, Queens, and Staten Island). As of May 2nd, 2020, Queens and Brooklyn were the top two counties in the United States for the total number of COVID-19 patients and number of deaths [2]. Interestingly, these counties, including the Bronx, had a higher per capita death rate due to COVID-19 than Manhattan [3]. There are several reasons to be considered. First, there are higher proportions of racial/ethnic minorities residing in these boroughs as compared to Manhattan. These populations tend to have higher rates of poverty and lower years of completed education, which may lead to the lower overall awareness of preventative measures as well as reduced access to healthcare [3]. The number of beds per 100,000 population was lower in these boroughs, especially Brooklyn (214 beds) and Queens (144 beds) when compared to Manhattan (534 beds) [3]. Third, location of care may contribute to difference in mortality. All these factors could affect higher mortality rates outside of Manhattan and our data suggest the government and hospitals should gather and allocate greater medical resources, especially for community hospitals in the area which has relatively fewer hospital beds per 100,000 population, during the pandemic of COVID-19. A previous study showed the comparison of mortality between NYC hospitals and non-NYC teaching hospitals resulting in higher mortality in the NYC cohort. Patient cohorts were similar except for more racial diversity in NYC patients, and NYC patients appeared to be sicker on admission [5].

Our data shows granular insights. Even after IPTW adjustment or multivariable adjustment for patient age, race/ethnicity, and comorbidities, patients in community hospitals had significantly higher mortality than those in teaching hospitals, which prompts us to consider that the hospital status and setting may play an important role in outcomes during the COVID-19 pandemic. During the first initial surge of COVID-19 during March/April 2020, hospitals attempted to accommodate the increasing number of patients requiring intensive care by expanding ICU beds and medical staff. However, ICU beds at Mount Sinai Queens and Brooklyn remained inadequate to meet the needs of patients admitted to these two hospitals, even though these hospitals doubled the number of ICU beds during the pandemic (from 37 to 79). It is consistent with higher in-hospital mortality rates among patients without ICU stay or intubation and implies that there was a shortage of supply which might have been one of the factors of high mortality rates at community hospitals. Moreover, our findings remained robust even after adjustments including endotracheal intubation, indicating that disease severity at the different hospital locations is unlikely to be the primary reason for the differences in mortality. The overall mismatch between the high number of COVID-19 patients in Brooklyn and Queens and the low number of per capita medical and ICU beds as well as the relative paucity of medical resources in community hospitals, make it difficult for hospitals to rapidly adapt to the evolving nature of the COVID-19 pandemic and hence contribute to the higher mortality rates seen in community hospitals.

A previous study showed that teaching hospitals tended to have the lowest mortality for hospitalized COVID-19 patients [6] and our study demonstrated that patients transferred from community hospitals to teaching hospitals had better survival than those who were treated in community hospitals only. Our data suggests community hospitals should consider transferring patients with COVID-19 to teaching hospitals to avoid excessive death rates especially during the pandemic of COVID-19 which causes an imbalance of patients’ populations and hospital beds.

Our study has important public health and policy implications. Early on during the pandemic, policymakers must quickly assess and determine which county/region will be most affected in terms of case burden and in turn will require more medical resources, based on the characteristics of residencies and hospital beds, especially ICU capacity. This will be crucial for the follow-up phases of the pandemic and our results suggest that more resources should be provided to community hospitals to alleviate the disparities in mortality [7]. However, we did not assess the quality of care provided in each hospital, which can contribute to outcomes. Thus, the multifactorial approach that combines financial support of community hospitals during the pandemic with continuous improvement of quality of team care across the entire care continuum can contribute to an improvement of care in community hospitals. In addition, government policies such as social distancing and stay-at-home orders to decrease the peak number of infected patients need to be continued to avoid a large number of deaths [8].

Our study has several limitations. Our study included data only from a single healthcare system in NYC, hence reducing the generalizability of our findings to other populations and healthcare systems. Despite fully adjusting for available patients’ baseline characteristics such as age, race/ethnicity, comorbidities, and vital signs as well as endotracheal intubation, remaining residual and unmeasured confounding factors including differences of socioeconomic status between each hospital location, could limit our causal interpretation. One of such factors could be the criteria for which patients were deemed suitable for transfer between community hospitals and teaching hospitals because this information was not readily available. Also, the thresholds for admission might be different between teaching hospitals versus community hospitals given the fact that elderly patients were likely to be admitted in community hospitals. The severity of illness progression upon admission must have varied however it was limited to vital signs at admission in the analysis. And finally, the availability of palliative or long-term care at teaching and community hospitals could affect in-hospital mortality, though we did not have access to this information.

In conclusion, patients who were exclusively cared for at community hospitals had higher mortality rates than those admitted to teaching hospitals, suggesting hospital status and settings might contribute to the differences in patients’ outcomes during the COVID-19 pandemic.

## Supplementary Information

**Additional file 1: Supplemental Lists 1.** The dependent variables to create propensity score for inverse probability treatment weight analysis to adjust hospital factors. **Supplemental Lists 2.** The dependent variables to create propensity score between patients hospitalized in community hospitals versus transfer patients. **Supplemental Table 1.** In hospital mortality, rates of ICU admission and intubation among patients admitted to teaching, community hospitals and transferred from community to teaching hospitals. **Supplemental Table 2.** In hospital mortality stratified by race. **Supplemental Table 3.** Age information of patients admitted to teaching, community hospitals and transferred from community to teaching hospital stratified by race. **Supplemental Figure 1.** Standardized mean difference in each comparison after inverse probability treatment weight adjustments. **Supplemental Figure 2.** Standardized mean difference after IPTW adjustments, transfer versus community hospitals.

## Data Availability

The datasets used and/or analyzed during the current study available from the corresponding author on reasonable request.

## Abbreviations

COPD: Chronic obstructive pulmonary disease
COVID-19: Coronavirus disease 2019
CI: Confidential interval
HIV: Human immunodeficiency virus
HR: Hazard ratio
IPTW: Inverse probability treatment weighted
SARS-CoV2: Severe acute respiratory syndrome coronavirus 2
