# Supplementary material for: Hospital factor and prognosis of COVID-19 in New York City, the United States of America: insights from a retrospective cohort study
Source: BMC Health Serv Res. 2022 Feb 8;22:164. doi: 10.1186/s12913-022-07570-w (PMC8826663; doi:10.1186/s12913-022-07570-w)
Supplement: Supplementary file 1 — Additional file 1: Supplemental Lists 1. The dependent variables to create propensity score for inverse probability treatment weight analysis to adjust hospital factors. Supplemental Lists 2. The dependent variables to create propensity score between patients hospitalized in community hospitals versus transfer patients. Supplemental Table 1. In hospital mortality, rates of ICU admission and intubation among patients admitted to teaching, community hospitals and transferred from community to teaching hospitals. Supplemental Table 2. In hospital mortality stratified by race. Supplemental Table 3. Age information of patients admitted to teaching, community hospitals and transferred from community to teaching hospital stratified by race. Supplemental Figure 1. Standardized mean difference in each comparison after inverse probability treatment weight adjustments. Supplemental Figure 2. Standardized mean difference after IPTW adjustments, transfer versus community hospitals. [file 12913_2022_7570_MOESM1_ESM.docx]

**Supplemental Lists 1:** The dependent variables to create propensity score for inverse probability treatment weight analysis to adjust hospital factors

The hospital factor where patients were treated (community hospitals, teaching hospitals, transfer), age, sex, race, asthma, chronic obstructive pulmonary disease, hypertension, obesity, diabetes mellitus, chronic kidney disease, human immunodeficiency virus, cancer, atrial fibrillation, heart failure, alcoholic/non-alcoholic liver disease, body mass index, vitals.

**Supplemental Lists 2:** The dependent variables to create propensity score between patients hospitalized in community hospitals versus transfer patients

Age, body BMI (normal [BMI 18.5-25 kg/m^2^] versus overweight [BMI 25-30 kg/m^2^] or obese [BMI > 30 kg/m^2^]), sex, race, asthma, chronic obstructive pulmonary disease, hypertension, obesity, diabetes mellitus, chronic kidney disease, human immunodeficiency virus, cancer, atrial fibrillation, heart failure, alcoholic/non-alcoholic liver disease, vitals, intensive care unit admission, and intubation

BMI: body mass index

Supplemental Table 1: In hospital mortality, rates of ICU admission and intubation among patients admitted to teaching, community hospitals and transferred from community to teaching hospitals

|  | Teaching hospitals  (n=3,520) | Community hospitals  (n=1,355) | Transfer (n=243) | P-Value |
| --- | --- | --- | --- | --- |
| Median LOS (days) | 5.6 | 6.0 | 10.2 | <0.001 |
| ICU admission, n (%) | 611 (17.4) | 174 (12.8) | 83 (34.2) | <0.001 |
| Intubation, n (%) | 374 (10.6) | 141 (10.4) | 63 (25.9) | <0.001 |
| Death, n (%) | 710 (20.2) | 618 (45.6) | 69 (28.4) | <0.001 |

LOS: length of stay, ICU: intensive care unit

Supplemental Table 2: In hospital mortality stratified by race

|  | Teaching  hospitals (n=4,742) | Community  Hospitals (n=1,471) | Transfer (n=296) | P-Value |
| --- | --- | --- | --- | --- |
| Death, n (%) |  |  |  |  |
| White | 19.6% | 50.5% | 25.5% | <0.001 |
|  | (200/1,019) | (206/408) | (14/55) |  |
| Black/Hispanic | 16.7%, | 37.4% | 21.6% | <0.001 |
|  | (413/2,473) | (279/745) | (37/171) |  |
| Other | 17.1% | 44.0% | 27.1% | <0.001 |
|  | (214/1,250) | (140/318) | (19/70) |  |

Supplemental Table 3: Age information of patients admitted to teaching, community hospitals and transferred from community to teaching hospital stratified by race

|  | Teaching  hospitals (n=4,742) | Community  Hospitals (n=1,471) | Transfer (n=296) | P-Value |
| --- | --- | --- | --- | --- |
| Age, mean (SD) |  |  |  |  |
| White | 63.6 (19.3) | 76.6 (12.9) | 67.1 (16.0) | <0.001 |
| Black/Hispanic | 60.5 (17.5) | 66.6 (15.0) | 57.6 (14.6) | <0.001 |
| Other | 60.9 (17.5) | 67.9 (14.1) | 59.7 (15.5) | <0.001 |

SD: standard deviation

Supplemental Figure Legends

Supplemental Figure 1: Standardized mean difference in each comparison after inverse probability treatment weight adjustments

COPD: chronic obstructive pulmonary disease, HIV: human immunodeficiency virus

Supplemental Figure 2: Standardized mean difference after IPTW adjustments, transfer versus community hospitals

COPD: chronic obstructive pulmonary disease, HIV: human immunodeficiency virus

Supplemental Figure 1

Supplemental Figure 2
